# Supplementary material for: The causal relationship between the human gut microbiota and pyogenic arthritis: a Mendelian randomization study
Source: Front Cell Infect Microbiol. 2024 Nov 26;14:1452480. doi: 10.3389/fcimb.2024.1452480 (PMC11629706; doi:10.3389/fcimb.2024.1452480)
Supplement: Supplementary file 1 [file DataSheet1.zip › mendelian test/ebi-a-GCST90027794.csv_leave_one_out.pdf]

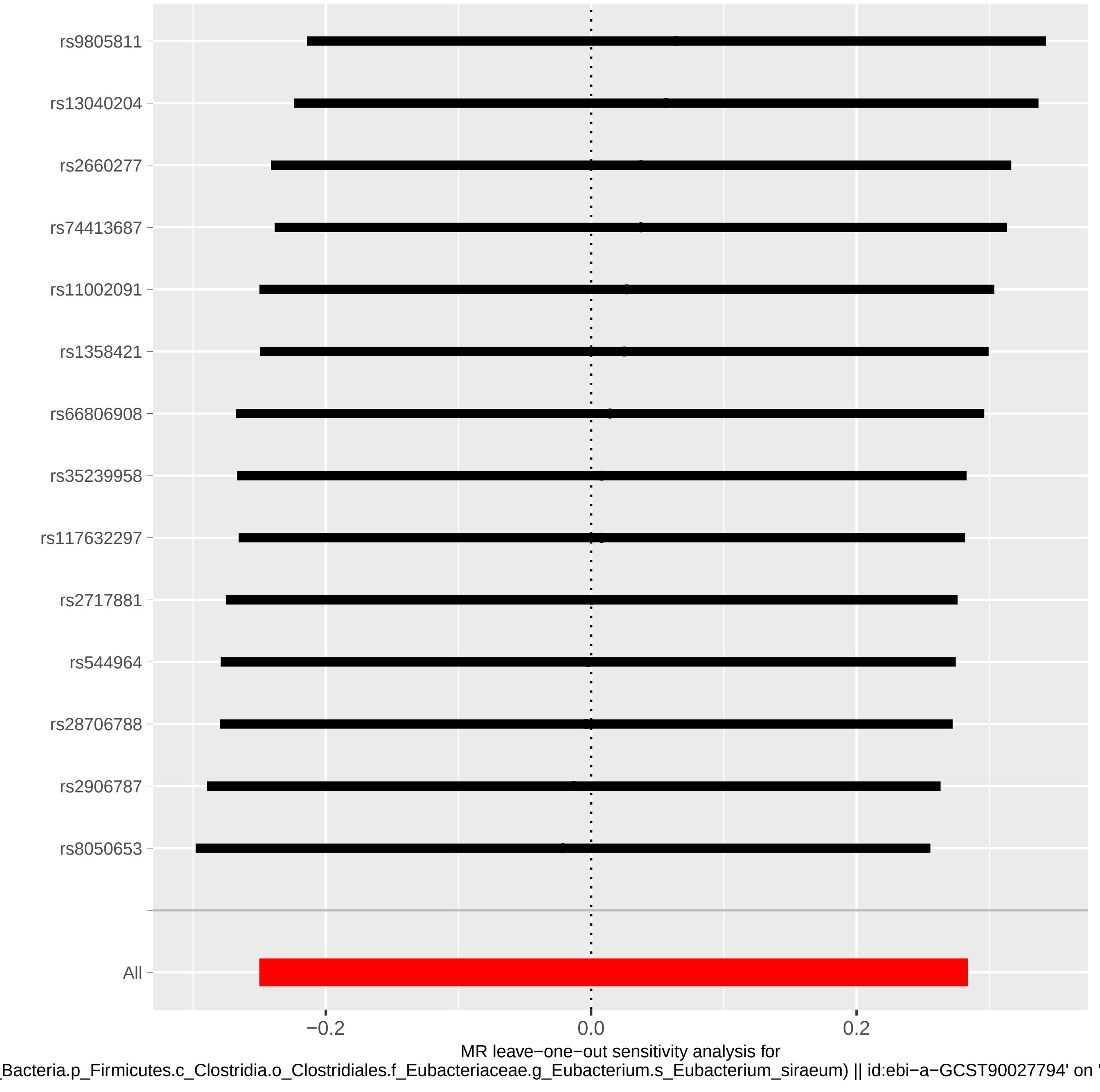

rs9805811
rs13040204
rs2660277
rs74413687
rs11002091
rs1358421
rs66806908
rs35239958
rs117632297
rs2717881
rs544964
rs28706788
rs2906787
rs8050653
All
−0.2
0.0
0.2
MR leave−one−out sensitivity analysis for
_Bacteria.p_Firmicutes.c_Clostridia.o_Clostridiales.f_Eubacteriaceae.g_Eubacterium.s_Eubacterium_siraeum) || id:ebi−a−GCST90027794' on '
